# Assessment of Dimethoate in Olive Oil Samples Using a Dual Responsive Molecularly Imprinting-Based Approach

**DOI:** 10.3390/foods9050618

**Published:** 2020-05-12

**Authors:** Raquel Garcia, Elisabete P. Carreiro, João Carlos Lima, Marco Gomes da Silva, Ana Maria Costa Freitas, Maria João Cabrita

**Affiliations:** 1 MED—Mediterranean Institute for Agriculture, Environment and Development, Departamento de Fitotecnia, Escola de Ciências e Tecnologia da Universidade de Lisboa, Universidade de Évora, Pólo da Mitra, Apartado 94, 7006-554 Évora, Portugal; afreitas@uevora.pt (A.M.C.F.); mjbc@uevora.pt (M.J.C.); 2 Centro de Química de Évora, IIFA, Universidade de Évora, Colégio L.A. Verney, 7000 Évora, Portugal; betepc@uevora.pt; 3 LAQV, REQUIMTE, Departamento de Química, Faculdade de Ciências e Tecnologia, Universidade Nova de Lisboa, 2829-516 Caparica, Portugal; lima@fct.unl.pt (J.C.L.); mdr@fct.unl.pt (M.G.d.S.)

**Keywords:** olive oil, dimethoate, molecularly imprinted polymers, magnetic responsiveness, photonic responsiveness

## Abstract

A new generation of advanced materials developed by molecular imprinting technology showing a stimuli-responsive functionality are emerging. The switchable ability to control the uptake/release of the target analyte by action of external stimulus combined with a remarkable selectivity and specificity, makes these functional materials very attractive for sample preparation purposes. In this work, the usefulness of a sample preparation tool for the selective enrichment/pre-concentration of dimethoate from olive oil spiked samples based on “tailor-made” dual responsive magnetic and photonic molecularly imprinted polymers as sorbents is explored. To achieve this goal, a smart molecularly imprinted polymer (MIP) possessing magnetic and photonic responsiveness was successfully synthesized, and its physico-chemical and morphological characterization was assessed. Further, the trace analysis of dimethoate in spiked olive oil samples was validated and successfully implemented using smart-MIPs as sorbents in the sample preparation step, with high recoveries (83.5 ± 0.3%) and low detection limit (0.03 µg·mL^−1^).

## 1. Introduction

Pesticides are used worldwide to manage agricultural pests. If the withdrawal period is not respected, they can persist until harvest time. The harmful effects for human health prompt the development of more sensitive and selective methodologies that enable an accurate quantification of pesticide residues in foodstuffs allowing to assess whether the maximum residue limits imposed by legislation are being met [1].

Commonly, the analysis of food samples encompasses an analytical workflow that includes a sample preparation step to isolate/pre-concentrate the target analytes. Indeed, even using the most advanced analytical techniques mainly based on chromatographic methods, direct injections of crude sample extracts are not a recommended practice. Moreover, the effect that some matrix components have on the suppression or enhancement of analyte ionization during their detection by mass spectrometry, which is well described in the literature, hampers an accurate quantification [1]. Thus, a sample preparation step is considered mandatory, particularly in samples of high complexity [2]. In recent years, very relevant advancements in sample preparation and purification processes emerged mainly due to the design and development of selective sorbent materials for application in food matrices, namely, molecularly imprinted polymers (MIPs) [2,3]. These polymeric materials are tailor-made to bind template molecules with high selectivity, even in the presence of structurally analogue molecules. In this synthetic procedure involving polymerization reactions, several entities are involved, like functional monomers, crosslinkers, porogen and the template molecule. Thus, MIPs are synthesized by copolymerization of functional monomers and cross-linkers in the presence of template molecules, using a porogen. In this process, a highly cross-linked polymer matrix possessing recognition cavities was produced. After removal of the template molecules, these recognition sites that are complementary in shape, size and spatial arrangement to the template molecules are able to rebind the template molecule [4,5]. In recent years, several works attempted to develop MIP-based sorbents aiming to enhance the selectivity of the sample preparation step on the trace analysis of pesticides in food samples. Therefore, application of MIPs in pesticides residues detection covers several classes, namely, organophosphorus pesticides (OPPs), triazines, carbamates, sulphonylurea and organochlorine pesticides (OCPs) [6,7,8,9,10]. Nowadays, an extensive research on the improvement of the sample preparation step based on MIP-based sorbents has been carried out. Sorbents are being designed and implemented, which in addition to being able to selectively retain the pesticide also exhibit a controllable release of the target analyte by means of external stimulus [11]. Indeed, the design of stimuli responsive molecularly imprinted polymers (SR-MIPs) as sorbents for solid phase extraction (SPE) seems to be a promising tool in sample pre-treatment, representing a step forward in the development of more advanced sample preparation techniques. These materials present “artificially generated” receptor sites in their three-dimensional structures enabling the selective “trapping” of the target analyte combined with stimuli-responsiveness by means of external specific stimulus [12,13]. The design of such kind of materials is challenging, particularly if several responsive elements are incorporated in the polymeric matrix, allowing to achieve more versatile functional sorbents. It should be accomplished in such a way that it enables each stimulus to be maximized without interference from others (orthogonality of the stimuli) while, simultaneously, maintaining the molecular recognition abilities. Owing to their properties, these materials are known as “smart” sorbents. Despite the usefulness and advantages that such SR-MIP based sorbents could bring to food sample preparation methodologies, their introduction in food analysis is in early stage of development for further validation and implementation [14]. Indeed, this topic still constitutes an open and promising area in the sample preparation field for target analysis, and the combination of appropriate stimuli could be explored to design SR-MIPs with enhanced properties for application in food analysis.

In recent years, our research group has been devoted to the implementation of solid phase extraction (SPE) techniques based on MIP sorbents, known as MISPE, for the trace analysis of pesticide residues in olive oil samples [15,16,17]. Other works have been also published mainly related to the use of MISPE for the selective detection of dimethoate and some congeners- methidathion and fenthion [18,19,20]. More recently, our investigations have explored the development of “host–guest” smart sorbents based on MIP technology with magnetic [21] and photonic [22] responsiveness for the selective enrichment of dimethoate from olive oil samples. 

Aiming to contribute to increase the knowledge and study the effectiveness of smart sorbents that integrate two responsive elements, a new functional polymeric material with a dual responsive character has been designed, synthesized and applied on the pre-treatment of olive oil samples. Dimethoate has been chosen as target pesticide since it is often used in Portuguese orchards to combat olive fly pest, one of the main enemies of olive orchards [23]. The application of pesticides in olive groves increases the likelihood of the presence of these residues in olive-derived foods. Due to the lipophilic character of these contaminants, it could determine their presence in olives and olive oils, if the withdrawal period was not respected [24]. Aware of the adverse health effects that this organophosphorus pesticide could cause to the consumers, which are mainly related with the inhibition of acetylcholinesterase (AChE) activity, causing acute toxicity; high persistency and bio accumulation with adverse effect on humans causing cancer, protein and endocrine disruption [25], European Union and the Codex Alimentarius Committee on Pesticide Residues and the Food and Agriculture Organization of the United Nations (FAO) have established maximum pesticide residue limits (MRLs) for olive oil [26,27]. Thus, the implementation of more advanced, straightforward, reliable and sensitive analytical methodologies that enables the quantification of trace levels of pesticide residues in complex matrices, like olive oil, is highly warranted. Altogether, the strategy used in this work encompasses some key innovations/improvements, mainly focused on the following points: (i) the design and development of “smart” functional MIPs based on core-shell magnetic-photonic MIP nanoparticles (DR-MIPs) for the selective trapping of dimethoate; (ii) an “on-off” integrated approach, timely controlled by the action of external magnetic and photonic stimulus, enables to gathering stirring/enrichment/separation into a single step and further photo-regulated release of the bounded pesticide. The dual magnetic and photonic responsiveness of DR-MIP is promoted by the incorporation in its structure of a Fe_3_O_4_-magnetic core and an azobenzene derivative as photochromic unit, respectively. Azobenzene photoisomerization has been known for many years and has been exploited to implement light-induced functionalities in synthetic materials giving them photo-inducing and controlling properties [28,29]. More recently, azobenzene derivatives have been explored as functional monomers on the synthesis of MIPs inducing a photo-responsive feature to those polymeric materials [30]. 

In this work, the use of a DR-MIP-based sorbent avoids tedious separation (filtration, centrifugation) steps and the sample-handling and time-consuming procedures are minimized, contributing to highlight the straightforward nature of this sample preparation methodology in comparison with the common MISPE technique. Xu and co-workers have used a similar strategy based on DR-MIP for the trace analysis of caffeine in complex samples [31]. However, in the scope of food safety issue, the present work represents an innovative approach. Furthermore, this methodology could be potentially applicable to a broad range of applications in food safety, since the synthesis of DR-MIPs could be tuned to other contaminants. 

## 2. Materials and Methods

### 2.1. Chemicals

Ferrous chloride (FeCl_2_•4H_2_O) (Panreac, Barcelona, Spain); ferric chloride (FeCl_3_•6H_2_O), 3-methacryloxypropyltrimethoxysilane (MPS), p-aminobenzoic acid (Acros), NaNO_2_ (Riedel-de Haen), phenol (Riedel-de Haen), Hydrochloric acid 37% (Chem-Lab), NaOH (José Manuel Gomes dos Santos), 1,1′-azobisisobutyronitrile (AIBN) and trimethylolpropane trimethacrylate (TRIM) were purchased from Sigma-Aldrich. Ammonium hydroxide (NH_4_OH, 25 wt %) was purchased from Manuel Gomes dos Santos. Acetonitrile (ACN), dimethylformamide (DMF), dimethyl sulfoxide (DMSO), tetrahydrofuran (THF), toluene for synthesis and acetic acid (AcOH) and methanol (MeOH) for MIP washing were obtained from Merck (Darmstadt, Germany). All the chemicals were used as received. 4-[(4-Methacryloyloxy) phenylazo]benzoic acid (MPABA) and magnetic precursor, Fe_3_O_4_@MPS were prepared and characterized according to our previous publication [21,22]. The water was distilled and purified by a Milli-Q system (Millipore, Bedford, MA, USA). High Performance Liquid Chromatography (HPLC) grade acetonitrile and methanol, n-heptane were purchased from VWR International S.A.S. (Fontenay-Sons-Bois, France). The analytical standard dimethoate (Dmt) was purchased from Sigma-Aldrich (Bellefonte, PA, USA). Prior to HPLC injection, all samples were filtered through 13mm syringe filters (w/0.45 μm PTFE membrane) (VWR, USA). The organic extra virgin olive oil was purchased from a local supermarket.

### 2.2. Instrumentation

ATR-FTIR spectroscopy analysis measurements (in the range 450–4000 cm^−1^) was performed on a PerkinElmer Spectrum Two IR spectrophotometer with Attenuated Total Reflection (ATR) accessory. Phenom ProX Desktop scanning electron microscope (SEM) instrument was used for characterizing the morphology of the synthesized imprinting systems, with an accelerating voltage set to 5–15 kV.

Hamamatsu L9588-06 Spot Light Source Ultra Violet (UV)-lamp was used coupled to a monochromator (Jobin Yvon, Horiba, H10 UV model), for the photoregulated uptake and release studies. Chromatographic measurements were performed using a HPLC Waters Alliance a 2695-series Separation Module equipped with Alliance Series Column Heater; detection was carried out using a photodiode array detector (2998 PDA Detector) (Waters, USA) in the range of 190–600 nm; LiChroCART C18 Purospher STAR reverse phase column (250 × 4.6 mm ID, 5 μm) (Merck Millipore, Germany). Empower 3 FR2 software was used for management, acquisition and treatment of data. Chromatographic conditions used in each assay were implemented in previous works of the team [21,22]. 

### 2.3. Synthesis of DR-MIP

The synthesis of DR-MIP involves a previous preparation of Fe_3_O_4_@MPS, which is based on a published procedure [21,22]. In detail, Fe_3_O_4_ (1.5 g) and toluene (50 mL) were added to a three-necked flask under nitrogen. After that, nanoparticles were sonicated for 1 h; the obtained solution was mechanically stirred, and MPS (5 mL) was added. The reaction was carried out at 50 °C with vigorous mechanical stirring overnight. Fe_3_O_4_@MPS nanoparticles were separated by using a magnet and then were washed with ethanol (2 × 30 mL), dichloromethane (3 × 30 mL) and then dried under vacuum at 40 °C during 5 h. Next, DR-MIP was prepared according to the following procedure. It must be noted that the reaction was carried out in the dark and under nitrogen atmosphere. In a 50 mL round-flask, 155 mg (0.5 mmol, 4 eq) of MPABA, 28.7 mg (0.125 mmol, 1 eq) dimethoate, 25 mL of ACN, 6 mL of DMF and 1 mL of DMSO were added. This mixture was sonicated at 0 °C for 30 min. Fe_3_O_4_@MPS (112 mg) and 0.96 mL (3 mmol, 24 eq) of TRIM were added to the mixture and sonicated at 0 °C for 1 h. Then, the mixture was allowed to warm up to room temperature. After 1 h, 100 mg (0.68 mmol, 5.4 eq) AIBN was added. The mixture was mechanically stirred at 70 °C within 24 h. Next, DR-MIP particles were separated from reaction liquid using a magnet. The removal of the template was provided by successively washing with MeOH, a mixture of MeOH:AcOH (9:1 (*v/v*)) followed by MeOH. Then, DR-MIP particles were dried under vacuum at 50 °C. DR-NIP (non-imprinted polymer) was synthesized using the same procedure but in the absence of the template (Dmt) in order to assess the non-specific binding (Section 2.5 and Section 3.2).

### 2.4. Physical and Morphological Characterization

ATR-FTIR spectroscopy was used for the physico-chemical characterization of the magnetic precursor- Fe_3_O_4_@MPS, and the molecular imprinting systems- DR-MIP and DR-NIP (non-imprinted polymer). The morphological characterization of the DR-MIP and DR-NIP was also achieved by scanning electron microscopy (SEM).

### 2.5. Evaluation of the Molecular Recognition of DR-MIP

To evaluate the suitability of DR-MIP for the adsorption of Dmt, the molecular recognition of this imprinting material has been assessed by photoregulated uptake and release of Dmt by the DR-MIP. Mostly, it involves switching on/off the UV light irradiation of the mixture containing the imprinting system and the target analyte, using a standard solution of Dmt in n-heptane (final Dmt concentration of 7.26 × 10^−6^ M). As referred above, tedious separation of the DR-MIP particles by centrifugation and filtration processes is avoided since DR-MIP exhibits a magnetic responsiveness. The following procedure was used: 50 mg of DR-MIP was added to a quartz spectrometer cuvette containing 1.5 mL of a standard solution of Dmt in n-heptane (the final Dmt mass was 2.0 μg). The resultant mixture was stirred and irradiated at 440 nm for 90 min. Subsequently, the supernatant and imprinted material were separated using a magnet. The supernatant was transferred into a vial, evaporated under nitrogen and the residue obtained reconstituted with 1 mL of acetonitrile and analyzed by HPLC/DAD. Methanol (2 mL) was added to the spectrometer cuvette containing the DR-MIP, followed by irradiation at 365 nm during 90 min. The times of irradiation have been chosen according to previous studies [22]. The mixture was again exposed to the magnetic field, and the liquid fraction was separated, evaporated to dryness under N_2_, reconstituted with 1mL of acetonitrile and analyzed by means of HPLC/DAD. The chromatographic conditions used were described in a previous work [21,22]. In order to access the degree of non-specific binding, a similar procedure was adopted for the corresponding DR-NIP. To evaluate the eventual differences between batch of DR-MIP in terms of molecular recognition behavior, three different 50 mg portions of three different batches of DR-MIP were tested according to the previous procedure. 

### 2.6. Implementation of the Analytical Methodology for the Selective Extraction of Dimethoate in Spiked Olive Oil Samples

**Figure 1:**
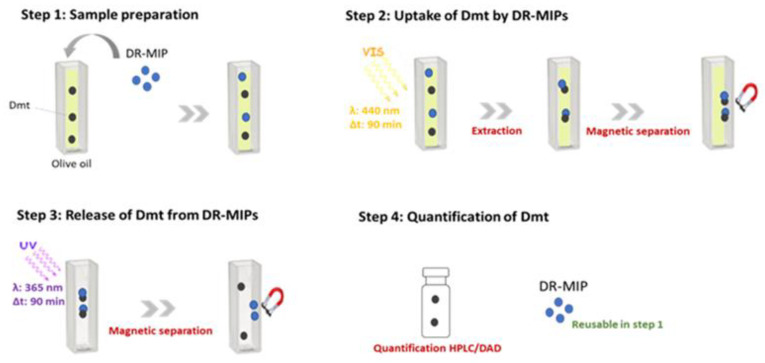
Schematic representation of the analytical methodology for the trace analysis of dimethoate (Dmt) in olive oil samples using core-shell magnetic-photonic molecularly imprinted polymer nanoparticle (DR-MIP) as sorbent.

In order to implement the sample preparation methodology based on DR-MIP as smart sorbent selective for Dmt, organic extra virgin olive oil has been used, and the absence of Dmt was confirmed by HPLC/DAD analysis. For this study, the spiking concentration was 2.0 μg·g^−1^, which corresponds to the maximum residue limits (MRLs) for this pesticide in olive products (Regulation (EC) No. 396/2005) [26,27]. The experimental procedure is outlined in Figure 1. Due to high complexity of olive oil sample, an improved version of the chromatographic method described in Section 2.2 has been used to ensure an efficient discrimination of the peak corresponding to the target analyte (Dmt) avoiding its possible coelution with matrix interferents. The chromatographic conditions used to perform these studies were the following: a binary mobile phase consisted of solvents A (water) and B (ACN) as follows: 25–100% B from 0 to 80 min, then 100% B from 80 to 85 min, followed by 100–25% B from 85 to 90 min and, after that, 25% B until 95 min; the flow rate was fixed at 0.4 mL·min^−1^ during the entire chromatographic process. The injection volume was 25 µL; a temperature of 25 °C; DAD detection was performed at 220 nm. All the determinations were conducted in triplicate and the average value calculated.

### 2.7. Screening Assays of the Reusability of DR-MIP

To evaluate the reusability of DR-MIP in the pre-concentration of dimethoate from olive oil spiked samples, the molecular imprinting-based sorbent has been continuously used on the workflow depicted in Figure 1. A total of 15 assays has been performed that corresponds to 15 utilizations of this functional sorbent. All the extracts were analyzed by means of HPLC-DAD as described in Section 2.6 and the concentration of Dmt assessed as well as the recovery rates. 

### 2.8. Experimental Validation (Calibration Curves/Repeatability)

The identification of dimethoate as well as the determination of calibration curves was performed as reported in previous works [21,22] The calibration curve of the Dmt (y = 3 × 10^7^ × −31,571) showed a linear range 0.45 µg·mL^−1^ and 3.15 µg·mL^−1^, a LOD and LOQ of 0.029 µg·mL^−1^ and 0.088 µg·mL^−1^, respectively, and a correlation coefficient (r) of 0.9998.

## 3. Results

### 3.1. Synthesis and Characterization of DR-MIP and DR-NIP

**Figure 2:**
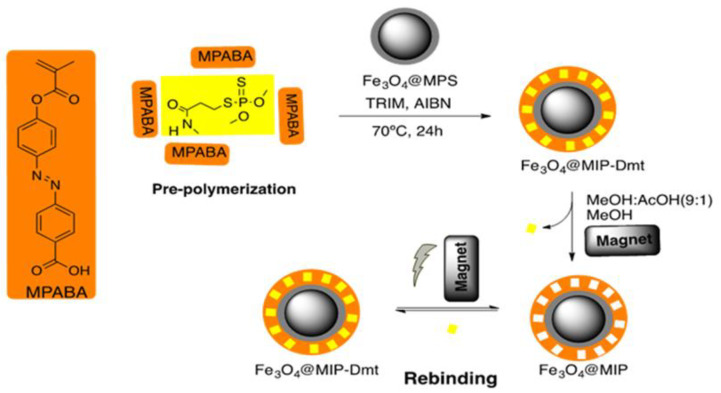
Synthetic pathway of the preparation of DR-MIP.

DR-MIP and DR-NIP were obtained by the surface-imprinting technique, as depicted in Figure 2. The synthesis of these imprinting materials involves the previous preparation of the magnetic precursor, Fe_3_O_4_@MPS, and the photo-responsive functional monomer-4-[(4-Methacryloxy) phenylazo] benzoic acid (MPABA). Both precursor and functional monomer were prepared and characterized according to previous works by our research team [21,22]. As usual, the synthesis of DR-MIP encompasses as first step a pre-polymerization that comprises the complexation between the functional monomer, MPABA, and the template Dmt in acetonitrile, dimethylformamide and dimethylsulfoxide, the porogen. The complexation was carried out at 0 °C in an ultrasonic bath, in the absence of light over 30 min and under nitrogen atmosphere. In our previous works [21,22], we stated, based on molecular modelling studies, the occurrence of a second complexation step before starting the thermal polymerization, which comprises the putative interactions between the cross-linkers and the preformed complex MPABA: Dmt. DR-NIP was prepared using the same synthetic strategy but without Dmt template. After the polymerization, both polymers (DR-MIP and DR-NIP) were washed with MeOH, MeOH:AcOH (9:1 (*v/v*)) and sequentially with MeOH, to remove the unreacted reagents and the template, in the case of the DR-MIP. Both, DR-MIP and DR-NIP were characterized using physico-chemical (ATR-FTIR) and morphological (SEM) techniques.

### 3.2. Characterization of DR-MIP and DR-NIP

#### 3.2.1. ATR-FTIR

**Figure 3:**
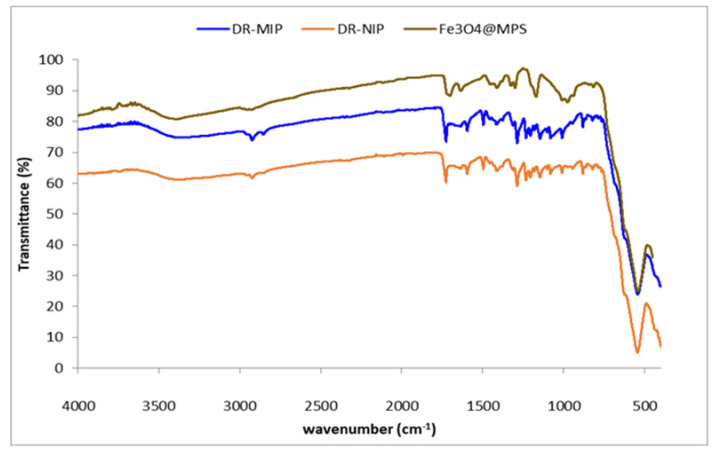
FTIR-ATR spectra of DR-MIP, DR-NIP (non-imprinted polymer) and Fe_3_O_4_@MPS.

Using the ATR-FTIR spectroscopy technique, it was possible to confirm the surface modification of the magnetic precursor, Fe_3_O_4_@MPS, by the imprinted polymer with photo responsive properties. The spectra of Fe_3_O_4_@MPS, DR-MIP and DR-NIP are showed in Figure 3. 

The absorption bands of the magnetic precursor, Fe_3_O_4_@MPS, were found in the spectra at 580 cm^−1^ (Fe-O), 1000 cm^−1^ (Si-O-Fe), 1300 cm^−1^ (C-O), 1640 cm^−1^ (C = C), 1722 cm^−1^ (C = O), 2964 cm^−1^ (C-H) and 3415 cm^−1^ (O-H). 

As expected, DR-MIP and DR-NIP spectra also show the absorption bands related to the presence of Fe_3_O_4_@MPS. Additionally, the presence of an absorption band at 1500 cm^−1^ characteristic of the diazo group (-N = N-) from the functional monomer (MPABA) corroborates the formation of the molecular imprinting systems (DR-MIP and DR-NIP). 

Thus, the analysis of ATR-FTIR spectra of the DR-MIP and DR-NIP confirms the presence of the magnetic precursor and the photo responsive monomer in their structure. 

#### 3.2.2. Morphological Characterization by SEM

**Figure 4:**
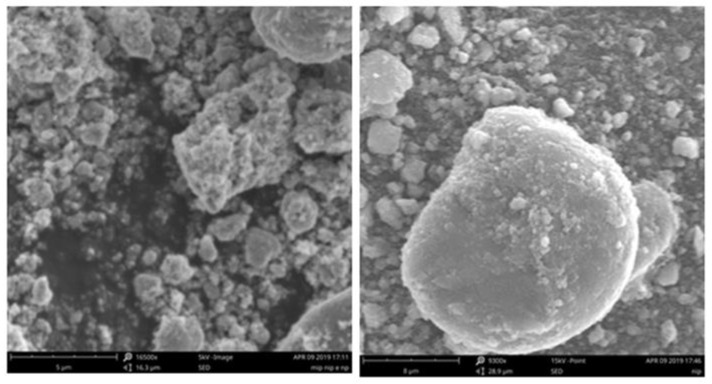
SEM images from DR-MIP (**left**) and DR-NIP (**right**).

The morphological topographies obtained by SEM of DR-MIP and DR-NIP are represented in Figure 4. It is possible to observe that DR-MIP has more irregular surface than the DR-NIP, probably due to the influence of the template (Dmt). The DR-MIP seems to be more porous and the DR-NIP to be more compact. Those morphological characteristics will have a profound impact on the rebinding properties of the imprinted and non-imprinted polymers. 

#### 3.2.3. Evaluation of the Molecular Recognition of DR-MIPs

The evaluation of the molecular recognition of DR-MIP is a preliminary assay on the development of selective sorbents for sample preparation procedures. Nevertheless, it is a crucial step since enables to ensure that the functional material displays molecular recognition abilities and that their application in pre-treatment of the sample does not compromise the successful implementation of a selective sample preparation methodology using DR-MIP as smart sorbent. Then, to address the suitability of DR-MIP as sorbent on the isolation/pre-concentration steps, an assay comprising the determination of the adsorption capacity of this functional sorbent has been performed using concentrations and conditions that mimic those which will be used on the implementation of the sample preparation methodology (Section 2.5). Thus, a concentration of Dmt similar to the MRL levels of this pesticide residue, legislated for olive oil products, has been used (Regulation (EC) No. 396/2005) [26,27]. The assays performed indicated that DR-MIP showed a retention capacity of 90.1 ± 0.3%, while the corresponding DR-NIP only presented a value of 49.2 ± 0.4%. The results prove that DR-MIP is appropriate to be used as sorbent in the pre-concentration step promoting a selective enrichment of the extract with the target pesticide (Dmt), showing an imprinting factor (IF) of 2.8.

The reproducibility of the molecular recognition of MIPs is a key topic on the implementation of a sample preparation methodology based on the use of MIPs as selective sorbents [1]. To evaluate this particular issue, three different batches of DR-MIPs were synthesized, and their molecular recognition ability was assessed. The assays performed, which use 50 mg portions of the three batches of DR-MIPs, show that the retention capacities vary in the range 89.6–90.4%. The data obtained indicates that the recognition behavior of DR-MIPs is reproducible even using polymeric material from different batches. 

#### 3.2.4. Implementation of the Analytical Methodology for the Selective Extraction of Dmt in Spiked Olive Oil Samples

The ultimate goal of this work is the implementation of a sample preparation methodology based on the use of DR-MIP as “smart” sorbent for the selective enrichment of Dmt from olive oil samples and further trace analysis of Dmt levels by means of HPLC/DAD. As depicted in Figure 1, in general, the implemented analytical methodology has some remarkable features, namely, (i) the use of an “intelligent” imprinting system in which the common physical robustness and thermal stability of MIPs are maintained as well as the pre-defined selectivity to the target molecule imparted by the molecular imprinting process; (ii) additional improvements on the straightforward photo-regulated uptake/release of the analyte through photoirradiation using light with appropriate wavelength, which can be manipulated precisely and rapidly; (iii) this is conjugated with magnetic properties, which allows a quick and easy separation, avoiding tedious separation, filtration and centrifugation steps. The methodology has been successfully implemented in organic olive oil samples spiked with concentrations of Dmt similar to the MRL enforced by the legislation, enabling to evaluate the efficiency of the extraction; a recovery rate of 83.5 ± 0.3% was obtained, with LOD and LOQ of 0.029 µg·mL^−1^ and 0.088 µg·mL^−1^, respectively. 

**Figure 5:**
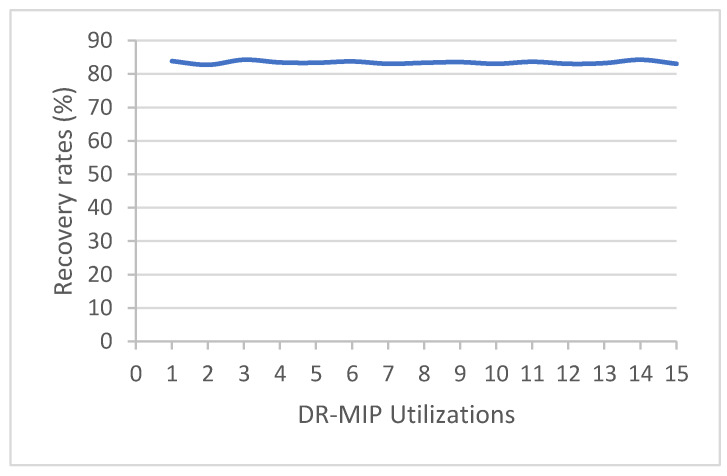
Reusability assays of DR-MIP.

A new trend in the development of sample preparation methodologies is to bestow on them an environmentally friendly character. Although, the most common solid phase extraction-based sorbents are not reusable, which constitutes one of the drawbacks related to sorption-based extraction techniques, MIPs can be reused for some extraction/pre-concentration cycles, which is highly advantageous in terms of costs and is an eco-friendly feature [14]. The reusability assays of DR-MIPs (Figure 5) show that this polymeric material does not have losses of recognition capacity for at least 15 cycles of dimethoate’s adsorption.

## 4. Conclusions

The combination of Molecular Imprinting Technology with stimuli-responsive elements is an ingenious synthetic process for the achievement of advanced functional materials that merge selectivity with a controllable uptake/release of the target compound. This is potentially promising as a “smart” tailor-made sorbent for sample preparation purposes [14]. In fact, DR-MIP designed in this work provides magnetic separation of Dmt from a complex matrix, like olive oil, followed by Dmt elution using UV-Vis irradiation, while keeping the selectivity. The whole system, gathering a selective enrichment, controlled separation and release of pesticide residues, can be reused, highlighting the straightforward, cost-effective and environmentally friendly features of this methodology. Thus, smart-MIPs merging an enhancement of selectivity with a controllable and switchable mode of action by means of combined use of specific stimulus appear as very promising, promoting several improvements on the trace analysis of dimethoate in a highly complex matrix, as olive oil. Despite this work being focused on the “case-study—Trace analysis of pesticide residues in olive oil based on nanosupported core-shell magnetic- photo DR-MIPs-approach”, their broad application to other edible vegetable oils widely consumed in other countries (e.g., soybean, hazelnut and sunflower oils, among others) would be reasonable. Furthermore, the synthesis of DR-MIPs could be tuned to other contaminants with relevance for the field of food safety, and this methodology could be extended to other applications.
